# CD64: An Attractive Immunotherapeutic Target for M1-type Macrophage Mediated Chronic Inflammatory Diseases

**DOI:** 10.3390/biomedicines5030056

**Published:** 2017-09-12

**Authors:** Olusiji A. Akinrinmade, Shivan Chetty, Adebukola K. Daramola, Mukit-ul Islam, Theo Thepen, Stefan Barth

**Affiliations:** 1 South African Research Chair in Cancer Biotechnology, Institute of Infectious Disease and Molecular Medicine (IDM), Department of Integrative Biomedical Sciences, Faculty of Health Sciences, University of Cape Town, 7925 Cape Town, South Africa; alex.akinrinmadex@gmail.com (O.A.A.); shivan.chetty@gmail.com (S.C.); dara.adebukola@gmail.com (A.K.D.); muksvipx1000@gmail.com (M.-u.I.); 2 Institute for Transfusion Medicine and Immunohematology and Blood Bank, University Hospital Magdeburg A.ö.R, 39120 Magdeburg, Germany; theophilus.thepen@med.ovgu.de

**Keywords:** CD64, immunotherapy, immunotoxins, human cytolytic fusion protein, dysregulated macrophage, chronic inflammatory disease

## Abstract

To date, no curative therapy is available for the treatment of most chronic inflammatory diseases such as atopic dermatitis, rheumatoid arthritis, or autoimmune disorders. Current treatments require a lifetime supply for patients to alleviate clinical symptoms and are unable to stop the course of disease. In contrast, a new series of immunotherapeutic agents targeting the Fc γ receptor I (CD64) have emerged and demonstrated significant clinical potential to actually resolving chronic inflammation driven by M1-type dysregulated macrophages. This subpopulation plays a key role in the initiation and maintenance of a series of chronic diseases. The novel recombinant M1-specific immunotherapeutics offer the prospect of highly effective treatment strategies as they have been shown to selectively eliminate the disease-causing macrophage subpopulations. In this review, we provide a detailed summary of the data generated, together with the advantages and the clinical potential of CD64-based targeted therapies for the treatment of chronic inflammatory diseases.

## 1. Introduction

**Figure 1:**
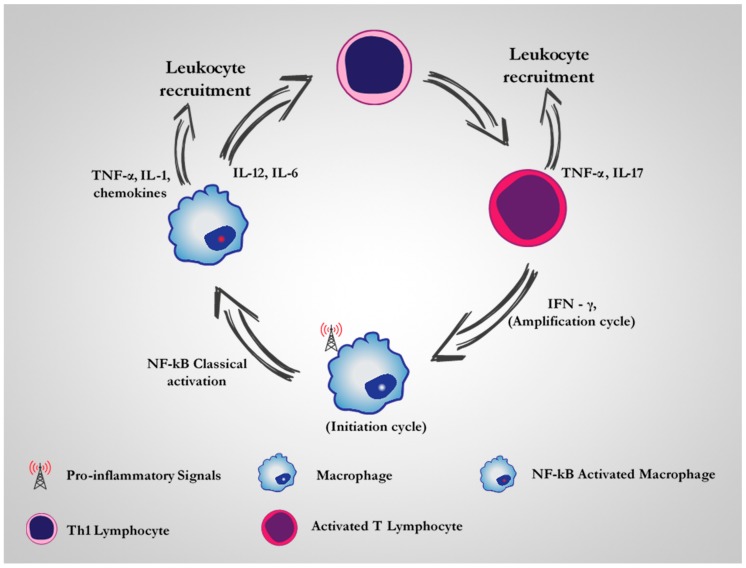
Cellular interplay during the initiation and amplification cycle of inflammation. During the early phase of inflammation, tissue-resident macrophages sense damage and become activated, herewith releasing signals that induce rapid recruitment of other immune effector cells. The stimulation and presentation of antigen to TH1 cells result a complex process which amplifies the phagocytic and destructive ability of macrophages.

A growing number of studies are increasingly confirming the involvement of chronic inflammatory processes in the pathogenesis of most infectious and non-infectious diseases [1]. More intriguingly, dysregulated or dysfunctional macrophages are now widely accepted as the major cellular players that drive disease initiation or maintenance processes [2]. Under normal circumstances, it is generally considered that there are several activated forms of macrophages at the onset of an inflammatory process. They have been frequently classified into two main groups, designated: (i) classically activated or pro-inflammatory macrophages (M1) and (ii) alternatively activated or anti-inflammatory macrophages (M2) [3]. Important to note is that this concept of M1 and M2 macrophage nomenclature which originated in the 1990s oversimplifies an extremely large spectrum of macrophage subpopulation [4,5,6,7]. Adhering to this simplified model, the M1 macrophages are activated in response to cytokines like tumour necrosis factor α (TNF-α) or interferon-γ (IFN-γ) and microbial products such as lipopolysaccharide (LPS) [8]. In a polarized response, M1 macrophages are aggressive against microbes and can engulf and digest affected cells much more readily and produce abundant pro-inflammatory cytokines such as TNF-α and pro-inflammatory mediators like nitric oxide (NO) [9]. On the other hand, M2 macrophages are promoted by signals such as IL-4, IL-13, glucocorticoids, IL-10, etc. and function in wound healing, tissue repair, and inflammation resolution [10]. Typically, the activation of M1 macrophages involves the induction of about 400 genes which result in the increased capacity to eliminate the inflammatory signal and recruit many other immune effector cells (e.g., neutrophils, T cells and natural killer cells) through the continuous production of pro-inflammatory cytokines and chemokines [11]. In essence, this process results in an amplification cascade that allows more macrophages to become activated and efficient in removing the inflammatory signal [12] (Figure 1). On the other hand, a prolonged activation or dysregulation of M1 activity can have damaging effects, such as septic shock, which can lead to multiple organ dysfunction syndrome (MODS) and in most cases death in 40% of patients [11,13]. In other situations, the development of chronic diseases such as rheumatoid arthritis (RA), chronic un-healing wounds, psoriasis etc. are associated with the persistence of pro-inflammatory macrophage activity [14]. For example, in patients with chronic RA, clear signs of M1 activity, such as high levels of pro-inflammatory or regulatory cytokines, growth factors (e.g., IL-1, IL-6, IL-13, TNF-α, granulocyte-macrophage colony-stimulating factor (GM-CSF)), chemokines, and chemoattractants (e.g., IL-8) are evident in the synovial membrane [15]. It is believed that these molecules interact with one another in a complex network that triggers a vicious cycle of pro-inflammatory signals resulting in chronic inflammation and joint destruction [16]. In particular, TNF-α is considered to be a key inflammatory cytokine in RA and its dysregulated secretion by macrophages is believed to be responsible for the continuous accumulation of leukocytes and maintenance of synovitis [17]. Importantly, the genes coding for TNF-α and most pro-inflammatory cytokines have been found to be under the control of NF-κB transcription factor [16]. The dysregulation of NF-κB pro-inflammatory signaling mechanisms has been suggested to be responsible for the development of most chronic inflammatory diseases [18]. Till date, there are presently no drugs available to cure or correct this pathological process.

The past two decades have seen the development of monoclonal antibody based drugs against pro-inflammatory targets such as tumor necrosis factor, interleukin-1 receptor, interleukin-6 receptor etc. These were reported to be clinically effective for the treatment of several inflammatory diseases [19]. For example, treatment of rheumatoid arthritis, may consist of blocking the effect of TNF-α using monoclonal antibodies such as infliximab, etanercept or adalimumab [20]. Infliximab, a human murine chimeric monoclonal antibody forms a strong non-disassociating bond with soluble and membrane-bound TNF-α to prevent the intracellular signalling that follows binding to its target receptor [21]. However, both therapeutic- as well as pathological implications have been revealed for anti-TNF-α therapy over the past few years. These include the activation of latent tuberculosis, pneumonia, histoplasmosis, candidiasis and aspergillosis and remain potential safety concerns to patients [22]. Also, not all patients show an initial response to infliximab since a good clinical response is dependent on significant level of expression of TNF-α in synovial biopsies prior to treatment. In addition, reports from clinical studies also show that the clearance of infliximab from the blood is associated with the return of clinical features in patients [21]. This may justify why current anti-cytokine therapies may require long term treatment to retain positive outcomes as they are not curative. The presence of populations of dysregulated pro-inflammatory macrophages—which are excessively producing the pro-inflammatory cytokines—could account for the relapse(s) seen with anti-cytokine therapies [20]. While approaches seeking to correct dysregulated NF-κB signaling cascades are promising, a balance between the benefit of such therapy and the drawback of interfering with normal cellular functions is still an issue of concern [23]. The natural tendency of macrophages to phagocytose foreign molecules has also been exploited by the encapsulation of cytotoxic agents into liposomes to allow non-targeted delivery into macrophage cell populations. This approach, despite its effectiveness in eliminating macrophage populations, possesses a significant health risk as it could compromise host immunity by destroying other phagocytic cells and preventing necessary macrophage response [24].

In recent years, the selective elimination of dysregulated M1 macrophages that maintain the uncontrolled secretion of pro-inflammatory cytokines have become the focus of the next generation of treatment. Such a novel strategy involves the design and use of immunotherapeutic agents that are capable of targeted delivery of cytotoxic or apoptosis inducing molecules (e.g., bacteria or plant toxins, human effector enzymes or synthetic small molecule compounds) into the cytoplasm of target M1 macrophages [20,25]. However, the development of these novel targeted technologies is quite challenging. The first perquisite for developing any such immunotherapeutic agent is the identification of a suitable surface molecule (most likely a receptor) specific for, or upregulated on, the target cell population. With macrophages this becomes even more complicated, most especially because macrophages are found throughout the body, are highly dynamic, and carry different physiological phenotypes at different time points, depending on the environmental tissue milieu or disease state [26]. Studies aimed at identifying clinically relevant surface receptors that will allow the discrimination between diseases causing M1 macrophages and M2 macrophages without off-target related adverse effects is crucial and remains an active area of investigation. This review will focus on CD64 as an attractive target for treatment of chronic inflammatory diseases mediated by dysfunctional M1-type pro-inflammatory macrophages.

## 2. CD64 as A Therapeutic Target for M1 Dysregulated Macrophages in Chronic Inflammatory Diseases

The cells of the monocyte phagocytic system naturally express a range of receptors including Fc receptors, complement receptors, cytokine receptors, chemokine receptors, mannose receptors, receptors for peptides, and small molecules etc. [10]. These receptors control activities such as growth, differentiation, activation, recognition, endocytosis, migration [27] and can be targeted for immunotherapeutic purposes by the use of an appropriate binding-ligand (e.g., peptides and antibodies). More recently, attention has been paid to Fc γ RI (commonly referred to as CD64) as a prime candidate to target these cells owing to its upregulation only under pro-inflammatory conditions [25]. Several in vitro, in vivo and ex vivo studies on patient samples have documented the upregulated levels of CD64 on activated macrophages at sites of chronic inflammation as a pre-requisite to validating its use and clinical suitability. The differential screening of M1-specific and M2-specific surface phenotypes using hCD64 transgenic mice and human macrophages after exposure to their respective inflammatory stimulus (M1: IFN-γ and LPS, M2: IL-4) has also been recently published [28]. In the study, eight receptors were found to be upregulated on murine M1 macrophages (Table 1) including CD64 and the co-receptor for LPS (CD14). Both CD64 and CD14 were upregulated on the human PBMC derived macrophage but downregulated on the M2 IL-4 stimulated macrophages. In contrast downregulated levels of the mannose receptor (CD206), haemoglobin scavenger receptor CD163, and macrophage galactose-type C-type lectin (CD301) were expressed on M1 macrophages but upregulated on the M2 macrophages [28]. These findings in addition to the strict myeloid cell distribution and ability to bind and rapidly internalize monomeric IgG, sets CD64 apart as a suitable target molecule which can be exploited for therapeutic purposes against dysregulated pro-inflammatory macrophages.

## 3. CD64: Background

Human CD64 is a transmembrane glycoprotein (72-kDa) that, with the Fc γ RII (CD32), and Fc γ RIII (CD16) receptors, comprises the large immunoglobulin (Ig) superfamily [29,30]. It binds monomeric IgG (for IgG1, IgG3 and IgG4) with high affinity in the nanomolar range (*K***_D_** ~10^−9^–10^−10^ M). This is particularly significant for the development of therapeutics for antibody-mediated autoimmune diseases. In mouse, the high affinity receptor (mCD64) binds monomeric mouse IgG2a with an affinity of *K*_A_ ~2 × 10^7^ M^−1^ [31]. When compared with other human Fc γ receptors, the affinity of human CD64 for monomeric IgG is 10–100 times higher than for the low-affinity Fc γ RII or Fc γ RIII family of receptors which interact poorly with monomeric IgG with binding affinity in the micro-molar range [32]. The high affinity of CD64 towards IgG demonstrates it pivotal role in initiating and activating cellular effector response even at low IgG concentrations [33]. Unlike CD64, other Fc γ receptors only internalize IgG complexes surrounding multivalent antigens. To better study the role and therapeutic potential of CD64, transgenic mice expressing human CD64 have been generated to enable the direct testing of human CD64-specific therapeutics in in vivo animal models [34].

### 3.1. CD64 Structure

**Figure 2:**
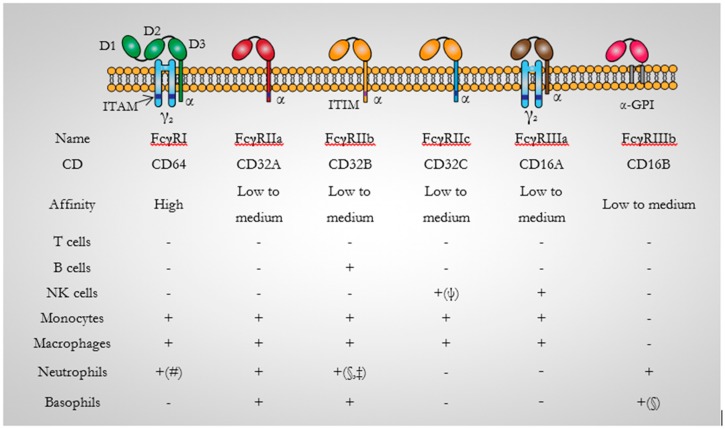
Diagrammatic representation of the human Fc receptors. There are five activating FcγRs: FcγRI, FcγRIIa, FcγRIIc, FcγRIIIa, FcγRIIIb, and one inhibitory Fc receptor; FcγRIIb. They all consist of an immunoglobulin-binding polypeptide chain with two Ig-like extracellular domains with the exception of FcγRI (CD64) which has three. An activating signalling cascade is mostly generated by the cross-linking of activating FcγRs by immune complexes which results in the phosphorylation of ITAMs on FcγRIIa and FcγRIIc. The FcR-γchain common to the Fc receptors is the only inhibitory FcγR. FcγRIIb also binds immune-complexed IgG and contain an ITIM in its cytoplasmic domain. ITAM: immunoreceptor tyrosine-based activation motif; γ_2_: dimer of FcRγ subunits; ITIM: immunoreceptor tyrosine-based inhibitory motif; GPI: glycosyl-phosphatidylinositol; NK cells: Natural Killer cells; +: indicates expression; (-): No expression; (#): only on activated Neutrophils; (§): Low; (‡): conflicting reports: (ψ): expressed only in 30% of humans [32,36].

To enhance the understanding of the functions and conformational changes upon interaction with IgG, the crystal structure of the extracellular domain of human CD64 has been determined to 2.65 Å resolution by molecular replacement [35]. Recently the crystal structure of human CD64 in complex with human Fc at 1.80 Å resolution has been resolved [30]. Structurally, α chains are reported to associate on the cell surface and consist of three extracellular Ig-like domains (D1, D2 and D3) which presents a V-like configuration, a transmembrane region, and a cytoplasmic domain [33] (Figure 2). Notably, three genes (FC γ RI A–C) have been identified for CD64 and are located on chromosome 1. One encoding the transmembrane region and two encoding the three Ig-like domains [33]. The hinge angles between the domains give CD64 a sea horse-appearance. A 35° and 120° hinge angle is reportedly configured between D1 and D2 and D2 and D3 domains respectively. These hinge angles are in comparison quite different especially when compared to the D1 and D2 domains of the human low affinity Fc γ receptors which present hinge angles ranging from 55° to 50° [35]. The high binding affinity of CD64 for IgG has also been associated with its unique hydrophobic pocket which perfectly accommodates Leu235 of human Fc at its surface. This precise conformational arrangement has been demonstrated by Kiyoshi et al., to be absent in other Fc γ receptors [30].

### 3.2. CD64 Signalling

CD64 plays a central role in macrophage antibody-dependent cellular cytotoxicity and clearance of immune complexes [37]. It has been shown that the internalization of immune complexes by CD64 is a two-step process. (i) The binding of monomeric ligand results in rapid internalization of the receptor to an endosomal compartment followed by rapid recycling to the cell surface. The ligand-triggered dissociation of CD64 from the cytoskeletal component; actin-binding protein (non-muscle filamin; ABP-280) is believed to provide a novel mechanism for regulating this receptor function. Unoccupied CD64 does not enter this internalization recycling pathway, showing that monomeric IgG binding itself is able and sufficient to initiate CD64 internalization. (ii) Cross-linking the occupied receptor causes retention of the ligand within the cell and subsequent degradation of the immune complexes, presumably in a lysosomal compartment. Intracellular accumulation of CD64-IgG occurs rapidly following cross-linking at a rate of 20–30% per min and essentially completes by 5 min [38].

### 3.3. CD64 Expression

CD64 is constitutively expressed on monocytes and macrophages and can also be induced on neutrophils with IFN-γ and G-CSF [39]. It is also expressed along with FcγRII on the myeloid derived cell lines HL-60, THP-1, and U937 [38]. Treatment with IFN-γ has been shown to up-regulate CD64 expression in U937 cells by 4–5-fold to a level of 60,000 receptors per cell [40]. Cytokine stimulation induces rapid clustering of CD64 on the cell membrane to facilitate rapid binding and internalization of immune complexes, including the de novo protein expression of new CD64 molecules to facilitate complex binding [41]. This in essence contributes to an inflammatory response by triggering release of TNF-α and IL-6, superoxide production, antigen presentation to T cells or lysis of antibody coated cells [39].

### 3.4. CD64-Specific Antibodies

The development of antibodies against CD64 has allowed for the generation and evaluation of targeted therapies as exemplified with monoclonal antibody (mAb) 197 which can specifically block phagocytosis and down-modulate CD64 expression on monocytes [42]. mAb 197 recognizes and binds two distinct epitopes on CD64. The first interaction is driven through its Fc domain to the Fc ligand-binding site on CD64 and the other through it Fab domain to an epitope outside the Fc ligand-binding site [42]. Clinically, the use of mAb 197 in a patient with chronic immune thrombocytopenia purpura (cITP) resulted in clinical improvement as mAb 197 infusion prevented FcγR mediated destruction of IgG-coated platelets [43]. As the murine origin of mAb 197 could potentially induce an immunogenic response, another murine derived CD64 specific monoclonal antibody (M22) that recognizes an epitope distinct from the natural ligand-binding site of IgG was developed by Guyre et al. and later humanized by Graziano et al. [40,44]. Humanization of the antibody was carried out by grafting its complementary determining regions (CDRs) onto human IgG1 constant domains, resulting in a full-length antibody (H22) which maintained binding specificity and high affinity for CD64 [44]. Using this antibody format, Heijnen and colleagues were able to develop early examples of antibody conjugates targeting human CD64 [45]. About a decade later, a single chain variable region fragment (scFv) version H22 with a lower molecular weight but same target specificity outside the normal Fc binding site was developed by De Kruif et al. [46]. This H22(scFv) has since been widely explored for targeted therapy as it does allow for efficient and rapid delivery of effector molecules to CD64 expressing cells in vitro and in vivo [47] irrespective of IgG saturation on the Fc domain of CD64.

## 4. CD64 Based Immunotherapeutic Studies in Chronic Inflammatory Diseases

In recent years, a novel class of CD64 directed immunotherapeutic agents have emerged for the treatment of macrophage-mediated chronic inflammatory diseases such as chronic cutaneous inflammation, rheumatoid arthritis, chronic diabetic wounds (CDW), etc. They have been accompanied by positive in vitro, in vivo and ex vivo results which make them highly promising candidates for clinical trials [48]. These novel immunotherapeutic agents are shown to successfully eliminate only the disease causing M1 pro-inflammatory and not M2 anti-inflammatory macrophage population even though both cell subtypes express CD64, albeit in low levels on M2 macrophages [28]. To better understand the mechanism of action (MOA) behind their profound selectivity for M1 macrophages, Hristodorov and colleagues hypothesized a possible difference in internalization and routing pathways by M1 and M2 macrophages for these CD64 directed immunoconjugates [28]. Data from their study revealed a significant increase in endosomal protease activity in both human and murine M2 macrophages which allowed for the destruction of the internalized immunoconjugate. A characteristics feature of their physiological status or function which enables the clearance and destruction of apoptotic cells during resolution of inflammation [49]. In contrast, a low endosomal proteolytic activity reported in the M1 macrophages is believed to be representative of the partial degradation needed by M1 macrophages to present antigens through the MHC pathway [50]. In consequence this is believed to limit the proteolytic degradation of the CD64 immunoconjugate after internalization and allow more time for translocation of the catalytic units into the cytosol where they execute their full cytotoxic activity [28]. Importantly, this selective killing of M1 pro-inflammatory macrophages through CD64 demonstrates several advantages for developing novel intervention strategies for chronic inflammatory conditions.

One clinical advantage includes a change in the tissue micro-environment in such a way that favours upregulation of M2-type anti-inflammatory macrophages. For example, Hristodorov et al., treated skin biopsies of chronic wounds from patients with atopic dermatitis and type II diabetes with the anti-CD64 immunotoxin (H22(scFv)-ETA’, reviewed later below). Analysis of the supernatants from the human skin biopsies for the presence of relevant cytokines before and after treatment revealed that the elimination of M1 macrophages significantly reduced the level of the pro-inflammatory cytokine IL-6, and induced an increased production of the anti-inflammatory cytokine IL-10 after 24 h [28]. As reported by the authors, this is clearly of advantage in that an anti-inflammatory environment could polarize newly infiltrating monocytes into developing into the M2 phenotype hereby maintaining a tissue micro-environment important for the resolution of inflammation. Indeed, upregulation of M2 macrophages will allow increase capacity to clear apoptotic neutrophils, and increased production of anti-inflammatory mediators responsible for stopping infiltration of other inflammatory effectors, such as T cells or dendritic cells [25]. A summary of some of these anti-CD64 based immunotherapeutics and their potential clinical relevance are described below.

### 4.1. Anti-CD64(H22)-Ricin A

In 2000, the first description of the successful use of CD64 as an immunotherapeutic target for the elimination of macrophages was demonstrated by Thepen and colleagues in a mouse model of cutaneous inflammation using CD64 monoclonal antibody (H22) chemically conjugated to the plant enzyme Ricin-A. As at that time, these classes of therapeutics were mostly prepared using heterobifunctional cross-linkers and belonged to the first generation of antibody drug conjugates. Ricin-A, the effector component, belongs to a group of “most toxic enzymes in nature” and are found as an abundant protein component of the *Ricinus communis* seeds (castor beans). Ricin-A inhibit protein synthesis as its mechanism of inducing cell death [51]. Its structure consists of an enzymatic polypeptide that upon translocation into the cytoplasm catalyzes the N-glycosidic cleavage of a specific adenine residue from 28S ribosomal RNA, to form a galactose (cell)-binding lectin. This enzymatic activity is responsible for rendering ribosomes containing depurinated 28S RNA incapable of protein synthesis [52].

Preclinical testing and results: Indeed, the use of the H22-R immunotoxin demonstrated the ability to selectively induce apoptosis with high specificity in activated pro-inflammatory macrophages based on their upregulated levels of CD64 without affecting resting low CD64 expressing macrophages in vitro. This was further demonstrated in vivo in a sodium lauryl sulfate (SLS) induced chronic cutaneous inflammation model in hCD64 transgenic mice. SLS directly applied on a shaved skin area (1.5 by 1.0 cm) for 10 consecutive days helped to induce chronic cutaneous inflammation after which intradermal injections of the H22-R and control were administered. The experiment showed resolution of cutaneous inflammation by 24 h as demonstrated by clearance of CD64 expressing macrophages in tissue sections. Also reported is the remarkable decrease in characteristic clinical features of local skin inflammation including skin temperature and vasodilation [25]. The results pioneered the promising approach of targeting CD64 to treating local cutaneous inflammation in patients. In the next experiment, the authors went ahead to demonstrate the ability of the immunotoxin (H22-R) to selectively eliminate inflammatory macrophages derived from rheumatoid arthritis patients. Indeed, the elevated levels of CD64 expressing inflammatory macrophages from synovial fluid of RA patients were markedly eliminated by the H22-R drug. By killing and removing this population of synovial fluid macrophages, authors saw a reduction in TNF-α and interleukin-1beta production which ultimately resulted in a significant decrease in cartilage-degrading activity of RA synovial tissue explants [53]. The selective elimination of inflammatory macrophages in an adjuvant arthritis model in hCD64 transgenic rats also confirmed the ability of CD64 directed immunotoxins to resolve inflammation and bone erosion in humans [54].

### 4.2. H22(scFv)-ETA’

**Figure 3:**
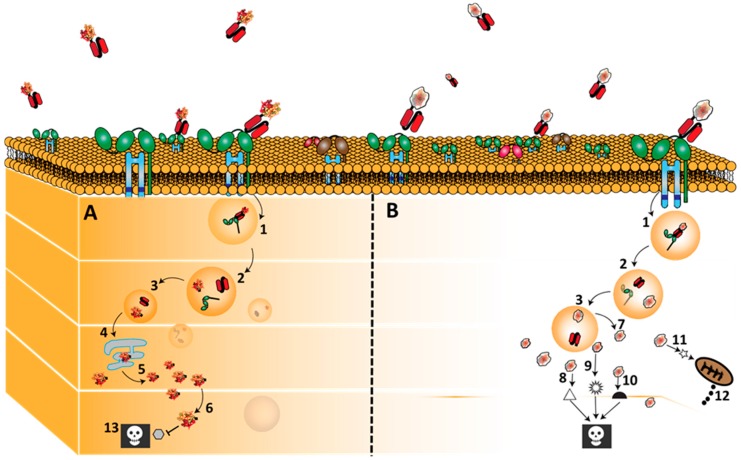
Binding, internalization and routing of CD64 directed bacteria immunotoxins (ETA’) and human cytolytic fusion protein (Granzyme B). H22 based fusion proteins bind CD64 and are internalize by receptor mediated endocytosis after which they are processed in the endosomal compartment. As mentioned earlier, the targeted killing of M1 macrophages by these fusion proteins is due to the differential post-internalization kinetics of these fusion proteins in M1 macrophages versus endosomal degradation in the M2-type macrophages. Once, internalized, a furin cleavage site separates the ligand from the effector molecules (1–3). ETA’ (**Panel A**) naturally contains a translocation domain by which it leaves the endosome and traffics through the endoplasmic reticulum into the cytosol (4–6) Here it enzymatically inactivates protein synthesis by ADP-ribosylation of EF-2,which subsequently leads to cell death (13). On the other hand, granzyme B based fusion proteins (**Panel** B) are often engineered to contain an adapter domain that allows their translocation into the cytosol (7) where they initiate apoptosis by cleaving; caspase dependent substrates (8), caspase independent substrates (9), BH3-only pro-apoptotic protein (Bid) (11) with subsequent cytochrome C release (12). Note (10): Some other intracellular apoptosis inducing pathway not yet identified.

Pseudomonas exotoxin A (ETA’) is the truncated version of the most toxic of all extracellular proteins (ETA) produced by pseudomonas aeruginosa bacteria [55]. Notably, immunotoxins based on this highly potent cytotoxic molecule (e.g., Moxetumomab pasudotox) are currently in clinical trials for the treatment of B cell malignancies and mesothelioma [56,57,58]. ETA’ based immunotoxins have been extensively studied and known to induce cell killing by catalysing ADP-ribosylation of a post-translationally modified histidine (diphthamide) on elongation factor 2 (EF-2) (Figure 3) [59]. EF-2, is responsible for the guanosine triphosphate-hydrolysis-dependent translocation of eukaryotic ribosomes during protein synthesis. ADP-ribosylated EF-2 is no longer able to mediate polypeptide chain elongation, by which ETA’-treated cells lose their ability to synthesize protein and ultimately die [60].

Preclinical testing and results: The selective cytotoxicity of CD64 based immunotoxins towards M1 murine (hCD64tg) and human macrophages was also confirmed by the resistance of M2 populations to H22(scFv)-ETA’ despite the expression of CD64 on both M1 and M2 macrophages. This specificity was further confirmed to be independent of the effector molecule as other effector enzymes tested conferred the same specificity towards M1 macrophages. However, it was observed that not all M1 macrophages are killed by the immunotoxins even at increasing concentration. This was explained by the assumption that among the M1 macrophages are some M2-like cells. This can be explained as a result of the non-uniformity in the life-cycle of M1 polarized state of isolated monocytic cell populations. Under in vivo conditions, intermediate polarized cells will be expected to be responsive to the changed M2- micro-environment created by the elimination of the M1 cells. In the same study, the elimination of M1 macrophages by H22(scFv)-ETA’ in a murine chronic cutaneous inflammation model resulted in a change in the inflammatory status towards an M2-biased microenvironment [28]. In a different study which took advantage of the ability of cross-linked CD64 to rapidly internalize IgG, bivalent H22(scFv)_2_-ETA’ were constructed and evaluated for increased uptake and cytotoxicity in CD64^+^ cells. As hypothesized, the H22(scFv)_2_-ETA’ fusion protein was more cytotoxic, killing IFN-γ stimulated CD64^+^ cell line (U637) at an IC_50_ value of 14 pmol L^−1^ when compared to monovalent H22(scFv)-ETA’ with an IC_50_ value of 140 pmol L^−1^. As would be expected, an increase in valency remarkably does improve the efficacy of CD64 based immunotoxin. Also, treatment with H22(scFv)_2_-ETA’ resulted in near complete clearance of CD64 expressing cells derived from punched skin biopsies in a chronic skin inflammation hCD64tg mouse model after 24 h [61].

### 4.3. Granzyme B-(H22)scFv

The third generation of anti-CD64 based fusion proteins are entirely made-up of components of human origin to prevent immunogenicity related clinical drawbacks [62]. One of such human cytolytic fusion proteins developed consist of the human granzyme B (Gb) effector molecule. Granzyme B (32-kDa), the most cytotoxic of all the human granzymes (others include A, H, K, M), was discovered and naturally produced in the cytoplasmic granules of cytotoxic T lymphocytes and Natural killer cells [63]. They are exceptional in their ability to induce apoptosis by proteolytically attacking several intracellular substrates including: multiple caspase family members (-3, -6, -7, -8, -9, -10), BH3-only pro-apoptotic protein (Bid), cell cycle regulatory proteins and/or kinase Cdc activation which makes them attractive for CD64 based therapy [64].

Preclinical testing and results: The use of a granzyme B based human cytolytic fusion protein directed against CD64 was first reported in 2008 by Stahnke et al., against acute myeloid leukaemia of subtypes M4 and M5 which are known to upregulate expression of CD64 [65]. In the study, the recombinant Gb-H22(scFv) based fusion protein showed functional proteolytic activity which was identical to unconjugated free granzyme with IC_50_ between 1.7 and 17 nmol/L in CD64^+^ U937 cells. Five years later, the group demonstrated the ability of gb-H22(scFv) to not only kill leukemic cells but also ex vivo differentiated human macrophages stimulated with IFN-γ and LPS [66]. The authors also identified for the first time the potential species-dependent cytotoxicity of human enzymes to their human substrate as neither Gb-H22(scFv) nor H22(scFv)-Angiogenin (discussed later below) could kill peritoneal macrophages from hCD64^+^tg mice. This discovery necessitated the use of primary cells from healthy individuals or patients with chronic inflammatory diseases to confirm in vivo animal studies and allow proper evaluation of CD64 based treatments being developed for clinical application. On the other hand, clinical application of granzyme B based therapies may be limited by the overexpression of its endogenous inhibitor (serpin B9) [67]. Serpin B9 (Protease Inhibitor-9) would normally protect immune cells present in a tumor micro-environment from T-cell or NK-cell misdirected granzyme B, but have also been found to be upregulated in several tumor types as an immune evading mechanism. About 5 years ago, Losasso and colleagues used computational approaches to identify several serpin B9 insensitive human granzyme B mutants [68]. Biological examination of these mutants showed that a R201K point-mutated granzyme B mutant demonstrated the highest cell killing potential in the presence of serpin B9 positive and negative cells [69]. Importantly, such granzyme B mutant would provide a significant advantage for the treatment of diseases in which dysregulated macrophages might upregulate serpin B9.

### 4.4. Granzyme M-(H22)scFv

The development of granzyme M based fusion protein for the treatment of chronic diseases takes on its well documented cell killing efficacy in cancer immunotherapy [70]. This includes its evaluation as an alternative granzyme which cannot be downregulated by serpin B9. Though the molecular mechanism of action of granzyme M (28 kDa) is still unknown, it is reported to exert its cytotoxic activity by cleaving peptide substrates after methionine or leucine [71].

Preclinical testing and results: The ability of Gm-H22(scFv) to kill activated CD64^+^ HL-60 cells have been reported with an IC_50_ between 1.2 and 6.4 nM. Importantly, the fusion protein had no impact on HL60 cells that had not been activated with IFN-γ which is beneficial for targeted treatments against chronic inflammatory diseases. The antigen-specificity of Gm-H22(scFv)-induced cytotoxicity was shown in a competitive binding assay with free H22(scFv), which inhibited cytotoxicity in a dose-dependent manner. [72].

### 4.5. H22(scFv)-Ang

Angiogenin (Ang) is a 14 kDa stress-activated enzyme and the 5th member of the human family of RNases [73]. Ang, through the process of angiogenesis, acts as a transcription factor for ribosomal RNA (rRNA), allowing the synthesis of molecules necessary for the formation of new blood vessels [74]. In addition, the ribonucleolytic activity of Ang allows it to hydrolyse cellular RNA (e.g., tRNA) [75,76]. To this effect, several angiogenin based immunoRNases have been designed and are able to trigger apoptosis by inhibiting protein synthesis via tRNA degradation upon delivery into the cytosol of target cells [77,78].

Preclinical testing and results: Since human enzymes do not possess the evolutionary adapted membrane translocation properties of bacteria enzymes which allows them to easily translocate into the cytosol, they are often accompanied with lower cytotoxicity because of lower cytosolic concentration. For an increased activity, human enzymes including angiogenin, are often re-engineered by mutating amino acid residues with key roles in the catalytic activity or adapted with synthetic translocation adapters. To this end, different Ang variants have been engineered and fused to the CD64-specific single-chain antibody fragment H22(scFv) to evaluate their cytotoxic activity in vitro. Notably, data from studies have shown modified Ang mutants to be more cytotoxic (IC_50_ of 0.6–6.7 nM) in targeted killing CD64^+^ HL60 cells when compared to wild-type H22-Ang conjugates (IC_50_ of 10 nM). The resulting IC_50_ values against human macrophages were from 43 to 153 pM for the Ang mutants and 287 pM for wild type construct. A G85R/G86R double amino acid mutation demonstrated the greatest cytotoxicity against activated CD64 cell populations due to its reduced (10^6^) affinity for its endogenous inhibitor (RNH1) [74,79]. Hence, these mutants can be used at low doses. In addition, the introduction of a sophisticated adapter sequence containing a membrane transfer peptide (MTD) in between an endosomal and cytosolic cleavable domains has also allowed an increase in the intracellular pool of Ang with a significant increase in cytotoxic activity to levels comparable to ETA’ based bacterial immunotoxins [80]. The potential combination of H22(scFv) and these Ang variants is expected to offer a powerful benefit for the treatment of M1 macrophage related chronic inflammatory conditions.

### 4.6. H22(scFv)-MAP

Human microtubule-associated protein tau (MAP tau) belongs to a group of microtubule targeting agents capable of inducing apoptosis in rapidly proliferating cancer cells [81]. To induce apoptosis, recombinant MAP tau binds to microtubule binding repeats on microtubules and by so doing, irreversibly enhance the polymerization of microtubules in a process that halts cell division and induces apoptosis in target cells [82]. Indeed, agents that target the mitotic phase of cell division to induce cell death have been the most successful in the treatment of cancer [83]. Example of such novel agents are the microtubule interacting small molecule toxins; e.g., auristatin E [83]. The use of MAP tau as the cytotoxic domain of human cytolytic fusion proteins also overcomes a major limitation associated with most human effector molecules. This includes cellular inactivation of human enzymes by endogenous inhibitors, to which no inhibitor has been reported for recombinant MAP tau. This makes MAP tau a very promising candidate for the development of immunotherapeutics against pro-inflammatory macrophages.

Preclinical testing and results: The expression and use of H22(scFv)-MAP confirmed the proliferation dependent cytotoxic activity of MAP tau by its ability to efficiently and selectively kill IFN-γ activated proliferating HL-60 cells. It was observed that there was no cytotoxicity of H22(scFv)-MAP to non-proliferating HL-60 cells stimulated with phorbol 12-myristate 13-acetate (PMA) to induce G0/G1 arrest and stop proliferation. Whereas apoptosis was strongly induced in IFN-γ stimulated HL-60 cells. In addition, H22(scFv)-MAP did not kill non-proliferating IFN-γ stimulated primary peritoneal macrophages isolated from hCD64tg mice and PBMC-derived human macrophages since they do not proliferate in vitro. This selectivity for proliferating M1 macrophages was also confirmed in situ. The identification of Ki67^+^ (cell proliferation marker) and CD64^+^ cells on inflamed skin tissues sections allowed authors to confirm the ability of CD64 expressing M1 macrophages to proliferate in vivo since this was a subject of debate. This selectivity by H22(scFv)-MAP for proliferating CD64^+^ M1 macrophages adds an additional level of targeted elimination for the treatment of chronic inflammatory diseases [84]. In addition, in vivo studies of other MAP tau based fusion constructs (e.g., EGF-MAP) shows that it is well tolerated at high doses [81].

## 5. Conclusions and Future Direction

In conclusion, targeting CD64 for the treatment ofdiseases is not new in literature. About two decades ago, bi-specific antibodies (BsAbs) were designed to evaluate CD64 directed treatment for patients with cancer. These BsAbs; MDX-H210 and MDX-447 were designed by chemical crosslinking of H22F(ab’), the humanized form of anti-CD64 mAb M22 to anti-HER2/neu) [85] and the anti-epidermal growth factor receptor respectively [86]. As reported in literature, MDX-H210 was successful in targeting monocytes and neutrophils in vivo and presented encouraging results when used in combination with GM-CSF in a trial treating patients with prostate cancer [41]. Clinical experience showed MDX-447 to be well tolerated in patients and active in cancers that have become refractory to all available therapies [87]. The report from a phase 1 trial combining H22 with Ki-4(anti-CD30) in patients with refractory Hodgkin lymphoma also presented 1 complete remission, 3 partial remissions, and 4 patients with stable disease out of a total panel of 10 patients [88]. However, these studies were hampered by issues of high toxicities [86,89], short half-lives and difficulties in generating true heterodimeric BsAbs from chemical cross-linking of antibody fragments. Though, these limitations can now be easily overcome with current state-of-the-art technologies. For example, chemical cross-linking of antibody fragments and its associated heterogenicity have been replaced by the genetic fusion of immunoreagents and the use of portable mini pumps to overcome short half-live problems [41].

In this paper, we summarized the early success and novel strategies recorded by CD64 targeted immunoconjugates in preclinical studies of chronic inflammatory diseases. Remarkably, this early pre-clinical success may have prompted evaluation of CD64 as a target for other macrophages or monocyte driven diseases including acute myeloid leukemia and leishmaniasis (Table 2). The involvement of dysregulated M1 macrophages in promoting chronic inflammation is clearly well established in literature and the reduction of this cell population, as depicted in this review, offers new opportunities for treatment of all associated diseases. Indeed, findings reviewed above demonstrate that removing dysregulated M1 macrophages from the vicious inflammatory cycle cuts inflammatory response and promotes an anti-inflammatory environment necessary for resolution of inflammation. Elimination of dysregulated M1 macrophages via CD64 offers an attractive tool for screening and development of novel treatment strategies. However, one important factor to remember as described above is that the selectivity for M1 macrophages by the CD64 fusion proteins is based on the probably high proteolytic degradation of the constructs in the M2-type anti-inflammatory macrophages. Furthermore, the ability to intervene in the chronic inflammatory cascade by modulating or re-polarizing M1-macrophages using for example CD64-directed immunomodulatory proteins, could offer great promise for future therapy and should be evaluated. For several decades, most well-known anti-inflammatory drugs like glucocorticoids, though very efficient have been accompanied by several drawbacks owing to their off-target systemic effect. The use of such immunomodulatory proteins targeted through CD64 at M1 pro-inflammatory macrophages may hence serve as another novel approach to correcting the M1/M2 imbalance and initiating the healing process for the treatment of chronic inflammatory disease. On the other hand, some preclinical hurdles remain to be solved in the context of recombinant immunotoxins. The removal of B and T cell epitopes from bacteria toxins to avoid immunogenicity remain a challenge as current approaches at deimmunizing ETA’ have often compromised its catalytic efficiency. Aside these limitations, the CD64-based human cytolytic fusion proteins described in this review seem to be well prepared for clinical applications and offer great promise for the treatment of M1-type macrophage mediated chronic inflammatory disease.

## Figures and Tables

**Table 1:** Differential screening of surface phenotype on M1 and M2-type macrophages by flow cytometry. Both murine and human macrophages were stimulated with INF-γ + LPS (M1) and IL-4 (M2). M1/M2 ratio > 0 = upregulated on M1 macrophages while M1/M2 < 0 = upregulated on M2 macrophages [28].

**Upregulated on M1 Macrophages**								
Murine	CD64	CD14	CD36	CD25	MHC I	CD39	MHCII	CD204
Human	CD64	CD14	CD16	CD284	CD80	CD273	-	-
**Upregulated on M2 Macrophages**								
Murine	CD206	CD273	CD284	CD301	CD11c	MOMA-1	MOMA-2	CD205
Human	CD206	CD200R	CD163	CD301	-	-	-	-

**Table 2:** Preclinical application of CD64 directed immunotoxins and human cytolytic fusion proteins. AML: Acute myeloid leukemia, CMML: Chronic myelomonocytic leukemia, AMML: Acute myelomonocytic leukemia.

Disease Model	Construct	Application	Remark	Reference
AML	H22(scFv)-ETA’	SCID mouse xenograft model for human AML	Potent anti-tumor activity against myeloid tumor cells, including a significantly prolonged the overall survival of AML xenograft animals	[90]
AML	Gb-H22(scFv)	In vitro and ex vivo	Specific binding to and elimination of CD64^+^ U937 cells as well as patient-derived CD64^+^ AML cells in vitro	[65]
AMML and CMML	Gb_mut_-H22(scFv)	In vitro and ex vivo	Induction of apoptosis in primary CD64^+^ AMML and CMML cells	[47]
AML, AMML, CMML, etc.	H22-Ang and mutants	In vitro and ex vivo	Induction of apoptosis in primary CD64^+^ leukemia cells isolated from patients	[79]
Leishmaniasis	H22(scFv)-ETA’ and H22-RiA	In vivo and ex vivo	Human: Selective killing of Leishmania infected monocytes. Mouse: arrest of cutaneous Leishmania model	[91]
Kidney transplantation	H22(scFv)-ETA’	In vivo	Preservation of renal integrity and function	[92]
AML and CMML	H22(scFv)-MAP	Ex vivo	Specific binding to and elimination of CD64^+^ leukemic blasts with no cytotoxicity towards healthy CD64^+^ PBMC-derived cells and macrophages	[82]

## References

[1] Hunter P. (2012). The inflammation theory of disease: The growing realization that chronic inflammation is crucial in many diseases opens new avenues for treatment. EMBO Rep..

[2] Wynn T.A., Chawla A., Pollard J.W. (2013). Macrophage biology in development, homeostasis and disease. Nature.

[3] Jain N.K., Mishra V., Mehra N.K. (2013). Targeted drug delivery to macrophages. Expert Opin. Drug Deliv..

[4] Martinez F.O., Gordon S. (2014). The M1 and M2 paradigm of macrophage activation: Time for reassessment. F1000prime Rep..

[5] Chávez-Galán L., Olleros M.L., Vesin D., Garcia I. (2015). Much more than M1 and M2 macrophages, there are also CD169^+^ and TCR^+^ macrophages. Front. Immunol..

[6] Murray P.J., Allen J.E., Biswas S.K., Fisher E.A., Gilroy D.W., Goerdt S., Gordon S., Hamilton J.A., Ivashkiv L.B., Lawrence T. (2014). Macrophage activation and polarization: Nomenclature and experimental guidelines. Immunity.

[7] Xue J., Schmidt S.V., Sander J., Draffehn A., Krebs W., Quester I., de Nardo D., Gohel T.D., Emde M., Schmidleithner L. (2014). Transcriptome-based network analysis reveals a spectrum model of human macrophage activation. Immunity.

[8] Utispan K., Koontongkaew S. (2016). Fibroblasts and macrophages: Key players in the head and neck cancer microenvironment. J. Oral Biosci..

[9] Obeid E., Nanda R., Fu Y.X., Olopade O.I. (2013). The role of tumor-associated macrophages in breast cancer progression (review). Int. J. Oncol..

[10] Kelly C., Jefferies C., Cryan S.A. (2010). Targeted liposomal drug delivery to monocytes and macrophages. J. Drug Deliv..

[11] Kasraie S., Werfel T. (2013). Role of macrophages in the pathogenesis of atopic dermatitis. Med. Inflamm..

[12] Helming L. (2011). Inflammation: Cell recruitment versus local proliferation. Curr. Biol..

[13] Monsel A., Zhu Y.-G., Genna S., Hao Q., Liu J., Lee J.W. (2014). Cell-based Therapy for Acute Organ InjuryPreclinical Evidence and Ongoing Clinical Trials Using Mesenchymal Stem Cells. J. Am. Soc. Anesthesiol..

[14] Valledor A.F., Comalada M., Lloberas J., Celada A. (2010). Macrophage Proinflammatory Activation and Deactivation: A Question of Balance. Adv. Immunol..

[15] Kinne R.W., Bräuer R., Stuhlmüller B., Palombo-Kinne E., Burmester G.R. (2000). Macrophages in rheumatoid arthritis. Arthritis Res. Ther..

[16] Simmonds R.E., Foxwell B.M. (2008). Signalling, inflammation and arthritis NF-κB and its relevance to arthritis and inflammation. Rheumatology.

[17] Shah B., Mayer L. (2010). Current status of monoclonal antibody therapy for the treatment of inflammatory bowel disease. Expert Rev. Clin. Immunol..

[18] Baker R.G., Hayden M.S., Ghosh S. (2011). NF-κB, inflammation, and metabolic disease. Cell Metab..

[19] Kotsovilis S., Andreakos E. (2014). Therapeutic human monoclonal antibodies in inflammatory diseases. Hum. Monoclon. Antib..

[20] Hristodorov D., Mladenov R., Huhn M., Barth S., Thepen T. (2012). Macrophage-targeted therapy: CD64-Based immunotoxins for treatment of chronic inflammatory diseases. Toxins.

[21] Maini R.N., Feldmann M. (2002). How does infliximab work in rheumatoid arthritis?. Arthritis Res. Ther..

[22] Derksen R., Bijlsma J. (2002). The treatment of chronic inflammatory diseases with monoclonal antibodies against tumor necrosis factor: Side effects, contraindications and precautions. Ned. Tijdschr. Voor Geneeskd..

[23] Tas S.W., Vervoordeldonk M.J., Tak P.P. (2009). Gene therapy targeting nuclear factor-κB: Towards clinical application in inflammatory diseases and cancer. Curr. Gene Ther..

[24] McCormick T.S., Stevens S.R., Kang K. (2000). Macrophages and cutaneous inflammation. Nat. Biotechnol..

[25] Thepen T., van Vuuren A.H., Kiekens R.C., Damen C.A., Vooijs W.C., van de Winkel J.G. (2000). Resolution of cutaneous inflammation after local elimination of macrophages. Nat. Biotechnol..

[26] Mosser D.M., Edwards J.P. (2008). Exploring the full spectrum of macrophage activation. Nat. Rev. Immunol..

[27] Burke B., Lewis C.E. (2002). The Macrophage.

[28] Hristodorov D., Mladenov R., von Felbert V., Huhn M., Fischer R., Barth S., Thepen T. (2015). Targeting CD64 mediates elimination of M1 but not M2 macrophages in vitro and in cutaneous inflammation in mice and patient biopsies. mAbs.

[29] Lu J., Sun P.D. (2015). Structural mechanism of high affinity FcγRI recognition of immunoglobulin G. Immunol. Rev..

[30] Kiyoshi M., Caaveiro J.M., Kawai T., Tashiro S., Ide T., Asaoka Y., Hatayama K., Tsumoto K. (2015). Structural basis for binding of human IgG1 to its high-affinity human receptor FcγRI. Nat. Commun..

[31] Bruhns P., Jönsson F. (2015). Mouse and human FcR effector functions. Immunol. Rev..

[32] Hogarth P.M., Pietersz G.A. (2012). Fc receptor-targeted therapies for the treatment of inflammation, cancer and beyond. Nat. Rev. Drug Discov..

[33] Raghavan M., Bjorkman P.J. (1996). Fc receptors and their interactions with immunoglobulins. Annu. Rev. Cell Dev. Biol..

[34] Mancardi D.A., Albanesi M., Jönsson F., Iannascoli B., Van Rooijen N., Kang X.Q., England P., Daëron M., Bruhns P. (2013). The high-affinity human IgG receptor FcγRI (CD64) promotes IgG-mediated inflammation, anaphylaxis, and antitumor immunotherapy. Blood.

[35] Lu J., Ellsworth J.L., Hamacher N., Oak S.W., Sun P.D. (2011). Crystal structure of Fcγ receptor I and its implication in high affinity γ-immunoglobulin binding. J. Biol. Chem..

[36] Bruhns P. (2012). Properties of mouse and human IgG receptors and their contribution to disease models. Blood.

[37] Dugast A.S., Tonelli A., Berger C.T., Ackerman M.E., Sciaranghella G., Liu Q., Sips M., Toth I., Piechocka-Trocha A., Ghebremichael M. (2011). Decreased Fc receptor expression on innate immune cells is associated with impaired antibody-mediated cellular phagocytic activity in chronically HIV-1 infected individuals. Virology.

[38] Harrison P.T., Davis W., Norman J.C., Hockaday A.R., Allen J.M. (1994). Binding of monomeric immunoglobulin G triggers Fc γ RI-mediated endocytosis. J. Biol. Chem..

[39] Hulett M.D., Hogarth P.M. (1998). The second and third extracellular domains of FcγRI (CD64) confer the unique high affinity binding of IgG2a. Mol. Immunol..

[40] Guyre P., Graziano R., Vance B., Morganelli P., Fanger M. (1989). Monoclonal antibodies that bind to distinct epitopes on Fc γ RI are able to trigger receptor function. J. Immunol..

[41] Van der Poel C.E., Spaapen R.M., van de Winkel J.G., Leusen J.H. (2011). Functional characteristics of the high affinity IgG receptor, FcγRI. J. Immunol..

[42] Wallace P.K., Keler T., Coleman K., Fisher J., Vitale L., Graziano R.F., Guyre P.M., Fanger M.W. (1997). Humanized mAb H22 binds the human high affinity Fc receptor for IgG (FcγRI), blocks phagocytosis, and modulates receptor expression. J. Leukoc. Biol..

[43] Ericson S., Coleman K., Wardwell K., Baker S., Fanger M., Guyre P., Ely P. (1996). Monoclonal antibody 197 (anti-FcγRI) infusion in a patient with immune thrombocytopenia purpura (ITP) results in down-modulation of FcγRI on circulating monocytes. Br. J. Haematol..

[44] Graziano R.F., Tempest P.R., White P., Keler T., Deo Y., Ghebremariam H., Coleman K., Pfefferkorn L.C., Fanger M.W., Guyre P.M. (1995). Construction and characterization of a humanized anti-γ-Ig receptor type I (Fc γ RI) monoclonal antibody. J. Immunol..

[45] Heijnen I., van Vugt M.J., Fanger N.A., Graziano R.F., de Wit T., Hofhuis F., Guyre P.M., Cape P.J.A., Verbee J.S., Winkel van de J.G.J. (1996). targeting to myeloid-specific human Fc γ RI/CD64 triggers enhanced antibody responses in transgenic mice. J. Clin. Investig..

[46] de Kruif J., Tijmensen M., Goldsein J., Logtenberg T. (2000). Recombinant lipid-tagged antibody fragments as functional cell-surface receptors. Nat. Med..

[47] Schiffer S., Rosinke R., Jost E., Hehmann-Titt G., Huhn M., Melmer G., Barth S., Thepen T. (2014). Targeted ex vivo reduction of CD64-positive monocytes in chronic myelomonocytic leukemia and acute myelomonocytic leukemia using human granzyme B-based cytolytic fusion proteins. Int. J. Cancer.

[48] Thepen T., Huhn M., Melmer G., Tur M., Barth S. (2009). Fcγ receptor 1 (CD64), a target beyond cancer. Curr. Pharm. Des..

[49] Ortega-Gómez A., Perretti M., Soehnlein O. (2013). Resolution of inflammation: An integrated view. EMBO Mol. Med..

[50] Mantegazza A.R., Magalhaes J.G., Amigorena S., Marks M.S. (2013). Presentation of phagocytosed antigens by MHC class I and II. Traffic.

[51] Parikh B.A., Tortora A., Li X.P., Tumer N.E. (2008). Ricin inhibits activation of the unfolded protein response by preventing splicing of the HAC1 mRNA. J. Biol. Chem..

[52] Lord J.M., Roberts L.M., Robertus J.D. (1994). Ricin: Structure, mode of action, and some current applications. FASEB J..

[53] Van Roon J.A., van Vuuren A.J., Wijngaarden S., Jacobs K.M., Bijlsma J.W., Lafeber F.P., Thepen T., van de Winkelet J.G. (2003). Selective elimination of synovial inflammatory macrophages in rheumatoid arthritis by an Fcγ receptor I–Directed immunotoxin. Arthritis Rheum..

[54] Van Vuuren A.J., van Roon J.A., Walraven V., Stuij I., Harmsen M.C., McLaughlin P.M., van de Winkelet J.G., Thepen T. (2006). CD64-directed immunotoxin inhibits arthritis in a novel CD64 transgenic rat model. J. Immunol..

[55] Wolf P., Elsässer-Beile U. (2009). Pseudomonas exotoxin A: From virulence factor to anti-cancer agent. Int. J. Med. Microbiol..

[56] Becker N., Benhar I. (2012). Antibody-based immunotoxins for the treatment of cancer. Antibodies.

[57] Wayne A.S., FitzGerald D.J., Kreitman R.J., Pastan I. (2014). Immunotoxins for leukemia. Blood.

[58] Weldon J.E., Xiang L., Zhang J., Beers R., Walker D.A., Onda M., Hassan R., Pastan I. (2013). A recombinant immunotoxin against the tumor-associated antigen mesothelin reengineered for high activity, low off-target toxicity, and reduced antigenicity. Mol. Cancer Ther..

[59] West S. (2000). Pseudomonas aeruginosa Exotoxin A: Structure/function, production, and intoxication of eukaryotic cells. Springer.

[60] Wilson B., Collier R. (1992). Diphtheria toxin and Pseudomonas aeruginosa exotoxin A: Active-site structure and enzymic mechanism. Curr. Top. Microb. Immunol..

[61] Ribbert T., Thepen T., Tur M., Fischer R., Huhn M., Barth S. (2010). Recombinant, ETA′-based CD64 immunotoxins: Improved efficacy by increased valency, both in vitro and in vivo in a chronic cutaneous inflammation model in human CD64 transgenic mice. Br. J. Dermatol..

[62] Berges N., Hehmann-Titt G., Hristodorov D., Melmer G., Thepen T., Barth S. (2014). Human cytolytic fusion proteins: Modified versions of human granzyme B and angiogenin have the potential to replace bacterial toxins in targeted therapies against CD64^+^ diseases. Antibodies.

[63] Bots M., Medema J.P. (2006). Granzymes at a glance. J. Cell Sci..

[64] Froelich C., Metkar S., Raja S. (2004). Granzyme B-mediated apoptosis-the elephant and the blind men?. Cell Death Differ..

[65] Stahnke B., Thepen T., Stöcker M., Rosinke R., Jost E., Fischer R., Tur M.K., Barth S. (2008). Granzyme B-H22 (scFv), a human immunotoxin targeting CD64 in acute myeloid leukemia of monocytic subtypes. Mol. Cancer Ther..

[66] Schiffer S., Hristodorov D., Mladenov R., Aslanian E., Huhn M., Fischer R., Barth S., Thepen T. (2013). Species-dependent functionality of the human cytolytic fusion proteins granzyme B-H22 (scFv) and H22 (scFv)-angiogenin in macrophages. Antibodies.

[67] Hehmann-Titt G., Schiffer S., Berges N., Melmer G., Barth S. (2013). Improving the therapeutic potential of human granzyme B for targeted cancer therapy. Antibodies.

[68] Losasso V., Schiffer S., Barth S., Carloni P. (2012). Design of human granzyme B variants resistant to serpin B9. Proteins Struct. Funct. Bioinform..

[69] Schiffer S., Hansen H., Hehmann-Titt G., Huhn M., Fischer R., Barth S., Thepen T. (2013). Efficacy of an adapted granzyme B-based anti-CD30 cytolytic fusion protein against PI-9-positive classical Hodgkin lymphoma cells in a murine model. Blood Cancer J..

[70] Susanto O., Trapani J., Brasacchio D. (2012). Controversies in granzyme biology. HLA.

[71] De Poot S., Bovenschen N. (2014). Granzyme M: Behind enemy lines. Cell Death Differ..

[72] Schiffer S., Letzian S., Jost E., Mladenov R., Hristodorov D., Huhn M., Fischer R., Barth S., Thepen T. (2013). Granzyme M as a novel effector molecule for human cytolytic fusion proteins: CD64-Specific cytotoxicity of Gm-H22 (scFv) against leukemic cells. Cancer Lett..

[73] Li S., Hu G.F. (2012). Emerging role of angiogenin in stress response and cell survival under adverse conditions. J. Cell. Physiol..

[74] Cremer C., Vierbuchen T., Hein L., Fischer R., Barth S., Nachreiner T. (2015). Angiogenin mutants as novel effector molecules for the generation of fusion proteins with increased cytotoxic potential. J. Immunother..

[75] Tello-Montoliu A., Patel J., Lip G. (2006). Angiogenin: A review of the pathophysiology and potential clinical applications. J. Thromb. Haemost..

[76] St Clair D.K., Rybak S.M., Riordan J.F., Vallee B.L. (1987). Angiogenin abolishes cell-free protein synthesis by specific ribonucleolytic inactivation of ribosomes. Proc. Natl. Acad. Sci. USA.

[77] Mathew M., Verma R.S. (2009). Humanized immunotoxins: A new generation of immunotoxins for targeted cancer therapy. Cancer Sci..

[78] Stöcker M., Tur M.K., Sasse S., Krüßmann A., Barth S., Engert A. (2003). Secretion of functional anti-CD30-angiogenin immunotoxins into the supernatant of transfected 293T-cells. Protein Exp. Purif..

[79] Cremer C., Braun H., Mladenov R., Schenke L., Cong X., Jost E., Brümmendorf T.H., Fischer R., Carloni P., Barth S. (2015). Novel angiogenin mutants with increased cytotoxicity enhance the depletion of pro-inflammatory macrophages and leukemia cells ex vivo. Cancer Immunol. Immunother..

[80] Cremer C., Hehmann-Titt G., Schiffer S., Melmer G., Carloni P., Barth S. (2015). Engineered Versions of Granzyme B and Angiogenin Overcome Intrinsic Resistance to Apoptosis Mediated by Human Cytolytic Fusion Proteins. Resist. Immun. Cancer Ther..

[81] Hristodorov D., Mladenov R., Pardo A., Pham A., Huhn M., Fischer R., Thepen T., Barth S. (2013). Microtubule-associated protein tau facilitates the targeted killing of proliferating cancer cells in vitro and in a xenograft mouse tumour model in vivo. Br. J. Cancer..

[82] Mladenov R., Hristodorov D., Cremer C., Gresch G., Grieger E., Schenke L., Klose D., Amoury M., Woitok M., Jost E. (2016). CD64-directed microtubule associated protein tau kills leukemic blasts ex vivo. Oncotarge.

[83] Akinrinmade O.A., Jordaan S., Hristodorov D., Mladenov R., Mungra N., Chetty S., Barth S. (2017). Human MAP tau based targeted cytolytic fusion proteins. Biomedicines.

[84] Hristodorov D., Mladenov R., Fischer R., Barth S., Thepen T. (2016). Fully human MAP-fusion protein selectively targets and eliminates proliferating CD64^+^ M1 macrophages. Immunol. Cell Biol..

[85] Van Ojik H., Repp R., Groenewegen G., Valerius T., van de Winkel J.G. (1997). Clinical evaluation of the bispecific antibody MDX-H210 (anti-FcγRI× anti-HER-2/neu) in combination with granulocyte-colony-stimulating factor (Filgrastim) for treatment of advanced breast cancer. Cancer Immunol. Immunother..

[86] Fury M.G., Lipton A., Smith K.M., Winston C.B., Pfister D.G. (2008). A phase-I trial of the epidermal growth factor receptor directed bispecific antibody MDX-447 without and with recombinant human granulocyte-colony stimulating factor in patients with advanced solid tumors. Cancer Immunol. Immunother..

[87] Curnow R.T. (1997). Clinical experience with CD64-directed immunotherapy. An overview. Cancer Immunol. Immunother..

[88] Borchmann P., Schnell R., Fuss I., Manzke O., Davis T., Lewis L.D., Behnke D., Wickenhauser C., Schiller P., Diehl V. (2002). Phase 1 trial of the novel bispecific molecule H22xKi-4 in patients with refractory Hodgkin lymphoma. Blood.

[89] Repp R., van Ojik H., Valerius T., Groenewegen G., Wieland G., Oetzel C., Stockmeyer B., Becker W., Eisenhut M., Steininger H. (2003). Phase I clinical trial of the bispecific antibody MDX-H210 (anti-FcγRI× anti-HER-2/neu) in combination with Filgrastim (G-CSF) for treatment of advanced breast cancer. Br. J. Cancer.

[90] Tur M.K., Huhn M., Jost E., Thepen T., Brümmendorf T.H., Barth S. (2011). In vivo efficacy of the recombinant anti-CD64 immunotoxin H22 (scFv)-ETA′ in a human acute myeloid leukemia xenograft tumor model. Int. J. Cancer.

[91] Van Weyenbergh J., Thepen T., Soares G., Khouri R., Barth S., Silva-Santos G., Costa J.M., Barral A., Barral-Netto M. (2013). 267: Challenging the Th1 paradigm: A detrimental role for IFN-γ and IFN-regulated CD64 in human leishmaniasis. Cytokine.

[92] Fet N.G., Fiebeler A., Klinge U., Park J.K., Barth S., Thepen T., Tolba R.H. (2012). Reduction of activated macrophages after ischaemia–Reperfusion injury diminishes oxidative stress and ameliorates renal damage. Nephrol. Dial. Transplant..

